# How Do COVID-19 Inpatients in the Denver Metropolitan Area Measure Up?

**DOI:** 10.1155/2020/8579738

**Published:** 2020-10-28

**Authors:** Paula J. Watts, Trevor Wojcik, Christina Baker-Sparr, Jason L. Kelly, Surit Sharma, Dmitriy Scherbak

**Affiliations:** ^1^HCA HealthONE-Sky Ridge Medical Center 10101 RidgeGate Parkway, Lone Tree, CO 80124, USA; ^2^Rocky Vista University, 8401 South Chambers Rd, Parker, CO 80134, USA; ^3^Critical Care and Pulmonary Consultants, 5200 DTC Parkway Suite 400, Greenwood Village, CO 80111, USA

## Abstract

**Background:**

Inpatient data for COVID-19 (SARS-CoV-2) afflicted inpatients remain sparse. Data are needed to create accurate projections for resource consumption as the pandemic continues. Published reports of inpatient data have come from China, Italy, Singapore, and both the East and West coasts of the United States.

**Objective:**

The objective is to present our inpatient experience with COVID-19. *Design, Setting, and Participants*. This is a retrospective study of 681 patients with laboratory-confirmed COVID-19 from six hospitals in the Denver metropolitan area admitted between February 18 and April 30, 2020. Clinical outcomes of patients discharged or expired by April 30, 2020, were analyzed. *Main Outcomes*. We compiled patient demographics, length of stay, number of patients transferred to or admitted to the ICU, ICU length of stay, mechanical ventilation requirements, and mortality rates.

**Results:**

Of the 890 patients with laboratory-confirmed COVID-19, 681 had discharged and were included in this analysis. We observed 100% survival of the 0–18 age group (*n* = 2), 97% survival of the 19–30 age group, 95% survival of the 31–64 age group, 79% survival of the 65–84 age group, and 75% survival of the 85 and older age group. Our total inpatient mortality was 13% (91 patients), rising to 29% (59 patients) for those requiring ICU care.

**Conclusions:**

Compared to similar reports from other metropolitan areas, our analysis of discharged or expired COVID-19 patients from six major hospitals in the Denver metropolitan area revealed a lower mortality. This includes the subset of patients admitted to the ICU regardless of the need for intubation. A lower ICU length of stay was also observed.

## 1. Introduction

In December 2019, COVID-19 (SARS-CoV-2) began its spread across the globe, resulting in the 13^th^ documented pandemic in human history. First identified in Wuhan, China, COVID-19 has spread worldwide with cases now in the millions in just six months [1, 2]. While drafting this report in May of 2020, there were still very little inpatient data regarding COVID-19. Early studies attempted to address hospital demographics, resource utilization, and mortality, but many did not exclude patients who remained admitted at the time of reporting [3–8]. Studies from Zhou et al. (Wuhan, China) [9] and Richardson et al. (New York City area, New York) [10] address this issue. In the United States, the Richardson et al. publication is the largest available inpatient dataset which includes 3,066 patients [10]. The state of Colorado constructed a model predicting length of stay (LOS), need for intensive care, ventilator requirements, and mortality in March 2020, before the Richardson et al. publication was available [10]. This model was based largely on assumptions from data outside of the United States; therefore, its utility for predicting resource utilization and patient outcomes in Colorado may be limited.

In order to compare our experience with other series from around the world, we evaluated inpatient data from 681 discharged patients from six hospitals in the Denver metropolitan area. This dataset includes inpatient and intensive care unit (ICU) length of stay, ICU admissions, mechanical ventilation days, total inpatient and ICU mortality, and readmissions. Our data show major differences from those collected by Zhou et al. (Wuhan, China) [9], Richardson et al. (New York City area, NY) [10], Bhatraju et al. (Seattle, WA) [7], and Grasselli et al. (Lombardy region, Italy) [8] and from the prediction models [11] used by the state of Colorado.

## 2. Methods

This retrospective cohort study was conducted with deidentified data from HCA's clinical database. Inpatient data abstracted from the HCA clinical data warehouse were aggregated for analysis and interpretation by the clinician authors.

### 2.1. Study Participants

The study sample comprised patients admitted to any of the six HCA-HealthONE hospitals in the Denver metropolitan area between February 18 and April 30, 2020, and who had a laboratory-confirmed COVID-19 diagnosis. COVID-19 testing was performed using a variety of nasopharyngeal swab PCR tests during this period. Patients who remained admitted as of April 30, 2020, as well as those transferred to another acute care hospital, were excluded from the final sample so that only those patients with a known discharge disposition or who had expired were considered for analysis. For patients who were readmitted during the study period, only data from the initial admission during which they received treatment for COVID-19 were included.

### 2.2. Variables of Interest

Patient demographic and clinical characteristics including age, sex, race, select baseline comorbidities, and smoking status were extracted. Comorbidities were categorized based on specific ICD-10 diagnosis codes documented in patient records. Primary clinical outcomes were discharge disposition, total length of stay, ICU admission, ICU length of stay, mechanical ventilation requirements, and mortality rates. Differences in these outcomes across age groups were also examined.

### 2.3. Statistical Analysis

Descriptive statistics were calculated using IBM SPSS Statistics Version 26 (SPSS Inc., Chicago, IL). Categorical variables were summarized as frequencies and percentages, while continuous variables were expressed as medians with interquartile ranges, due to the nonnormal distribution of the data. No imputation was made for missing data. Chi-square test and Cramer's V were used to analyze associations between different variables where appropriate.

## 3. Results

**Figure 1:**
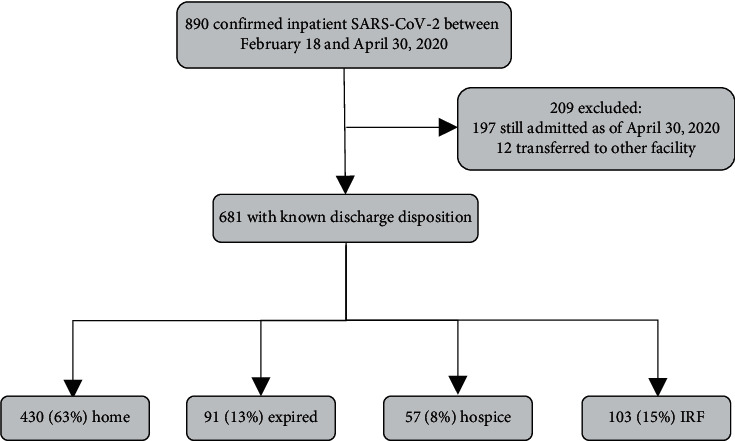
Study cohort. *∗*IRF = inpatient rehabilitation facility, which includes rehabilitation facility, skilled nursing facility, and long-term care hospital.

Of the 890 confirmed inpatient COVID-19 cases between February 18 and April 30, 2020, 209 were excluded. Excluded patients consisted of 197 who were still admitted at the time of data analysis and 12 who were transferred to an acute inpatient facility. We included 681 cases with definitive discharge data (Figure 1). Of those, a total of 49% (334) were female and 51% (347) were male. The median age was 64 years, with an interquartile range (IQR) of 50–78. Common comorbidities included hypertension, diabetes, and obesity. 24% (164) had one comorbidity, and 54% (370) had more than one comorbidity. 12% (80) had a history of chronic obstructive pulmonary disease. 15% (104) were former smokers, and 4% (30) were current smokers (Table 1). Relationships between gender, smoking history, obesity, and the presence of comorbidities were compared and analyzed with mortality, ICU stay, mechanical ventilation, and length of stay by assessing group differences within each variable (Table 2). Overall, though there were percentage differences between the groups, none were determined to be statistically significant except for the presence of comorbidities and mortality (Χ^2^ = 20.75, *p* < 0.001). Since there were large sample size differences between the comorbidity subgroups, Cramer's V was used following the chi-square test to measure the effect size of any statistical difference and was determined to be “small” (Cramer's V = 0.18, df = 2).

After stratifying patients by age group, only 2 patients were of age 18 and under; both discharged alive. Of the 19–30 age group, 3% (1) died, while 97% (29) were discharged alive. Of the 31–64 age group, 5% (17) died, while 95% (301) were discharged alive. Of the 65–84 age group, 21% (48) died, while 79% (185) were discharged alive. Of the 85 and above age group, 26% (25) died, while 75% (73) were discharged alive (Table 3).

Total inpatient mortality was 13% (91). The median LOS was 5.00 days (IQR, 2.79–9.83). Of those discharged alive, the median LOS was 4.75 days (IQR, 2.75–9.76), while the median LOS for those expired was 6.96 (IQR, 3.96–10.29). 30% (204) required ICU level of care. The total LOS for those requiring ICU was 6.82 days (IQR, 2.56–12.27). ICU mortality was 29% (59). Total patients requiring invasive mechanical ventilation was 17% (118). Median days on mechanical ventilation was 9.00 (IQR, 5.00–13.00). Mortality for those that received mechanical ventilation was 42% (49). Mortality of those that did not receive mechanical ventilation was 8% (42). Among the total patient population, there were 63% (430) discharged to home, 13% (91) expired, 8% (57) discharged to hospice, and 15% (103) discharged to either skilled nursing facility, inpatient rehabilitation facility, or a long-term care hospital. 13% (86) were readmitted (Table 4). We combined comparative data from select studies, which are illustrated in Table 5.

## 4. Discussion

As of May 3, 2020, the total cumulative case number in the world is 3,508,566. 1,158,041 are within the United States, 316,415 of which are in New York State, and 16,635 of which are in Colorado [2]. There have been 68,736 hospitalized in New York State and 2,793 in Colorado [2].

Inpatient data are sparse, coming from just a few published sources. Of previously available inpatient data, the typical COVID-19 inpatient worldwide is male (54–73%) [3, 5–7, 9, 10, 12] and has a length of stay between 4 and 12 days [5, 9, 10, 12]. Hospital mortality is 21–28% [9, 10, 12]. ICU mortality is 61–78% [7, 8, 10]. 64–86% of those admitted to the ICU required mechanical ventilation, and mortality for those ranges from 69 to 97% [7, 9, 10].

Compared to other available studies, we report decreased ICU LOS, mortality (both total and ICU), and ventilator days. Our median length of stay is 5.00 days, which is similar to that reported by Richardson et al. (New York City area, NY) but far less than reports from other metro areas with significant outbreaks [10]. ICU length of stay is similarly less than reports from other metro areas; however, this value was not available from Richardson et al. for comparison. Both our inpatient (13%) and ICU (29%) mortality are less than Richardson et al. [10], Zhou et al. (Wuhan, China) [9], Grasselli et al. (Lombardy, Italy) [8], and Bhatraju et al. (Seattle, WA) [7]. Time on mechanical ventilation (9 days) was shorter than other published data (range of 10–17 days) [3, 7].

Both our admission rate to the ICU and ventilator requirement are similar to or higher than those in other studies. Our ICU admission rate (30%) is greater than that predicted for Colorado as well as that reported by Richardson et al. (New York City area, NY) of 14% or Zhou et al. (Wuhan, China) of 26% [9, 10]. Among all hospitalized COVID-19 patients, 17% were intubated and placed on ventilator support, more than that noted by Richardson et al. of 12% and similar to Zhou et al. of 17% [9, 10].

There can be a number of explanations for our experience in our six Denver area hospitals. First, and possibly most significant, our average COVID-19 patient has fewer comorbidities. 54% of patients have more than one comorbidity, compared to 88% reported by Richardson et al. from the New York City area, NY [10]. 24% of our patients have only one comorbidity, compared to 6% in the Richardson et al. study [10]. 22% have no comorbidities, compared to 6% in the Richardson et al. study [10]. Our data show 23% of patients with a BMI of 30 or greater, while Richardson et al. report 42% [10]. In our population, patients without comorbidities have a 2% mortality which rises to 5.5% with the presence of one comorbidity and 21% with more than one comorbidity. Though the difference in mortality is significant depending on the overall number of comorbidities, we found no statistically significant difference between the comorbidities themselves. Our study is likely insufficiently powered for such analysis (Table 2).

Second, Colorado did not experience the resource constraints that were evident in Wuhan or New York City. We admitted a higher percentage of patients to the ICU (30%) than reported by Zhou et al. from Wuhan, China (26%) [9] or Richardson et al. from the New York City area, NY (14%) [10]. However, a lower percentage of our ICU patients required intubation (58%) compared to 64% reported by Zhou et al. [9] and 86% reported by Richardson et al. [10].

Colorado lagged behind other states and countries in terms of number of cases, likely a result of geographic location and distance from coastlines. We therefore had the benefit of their experience. Experimental therapies were also available, but the extent of their use or efficacy is not known at this time. Other preparations such as a timely stay-at-home order, stopping all elective surgeries to preserve equipment, and upstaffing of hospitals could certainly have improved outcomes. Interestingly, Colorado ranks near the bottom of the United States in terms of hospital beds per 1,000 persons at 1.9. For comparison, New York State has 2.7 [13].

COVID-19 is spread primarily by respiratory droplets [14]. The New York-Newark-Jersey City metro area has the highest population density in the United States with over 2,409.3 people per square mile, while the Denver metro area has an estimated 351.4 people per square mile [15, 16]. This factor may partly explain the increased transmissibility in the New York City area. It has been suggested that physical crowding may also increase the severity of disease because SARS-CoV-2 is different from other coronaviruses in that it can effectively take hold in either the upper or lower respiratory tract. Higher inoculum at initial exposure may facilitate particle travel to the lower respiratory tract, resulting in more profound illness [17].

COVID-19 data on climate and transmissibility speak against decreased transmissibility in the Denver area. A study by Wang et al. (unpublished data, 2020) investigated air temperature and relative humidity in 100 Chinese cities and 1,005 United States counties [18]. This analysis indicated higher temperatures and higher relative humidity may reduce the transmissibility of COVID-19 and is supported by other reports which have yet to be published [19, 20]. The climate in Denver, Colorado, was relatively dry and cold between February and April 2020. Furthermore, Denver is located at a significant elevation above sea level at 5,280 feet (about 1 mile or 1.609 kilometers), and the adjacent Rocky Mountains have even higher elevations. Traditionally, travel to high altitude has been associated with exacerbation of respiratory disorders [21].

While our data were obtained during peak incidence of COVID-19 in the United States, the clinical endpoint of inpatient mortality remains premature. A number of COVID-19 patients remain hospitalized at the time of data retrieval, and the pandemic is likely far from over. In this study, we excluded those that remained admitted to a hospital, while other studies did not [3–8, 12]. As a consequence, reported survival rates in contemporaneous studies may appear falsely higher. This study is limited to six hospitals in the Denver metro area, although these six do encompass a large portion of the city representing a wide spectrum of socioeconomic diversity. The 890 patients admitted to them represent about one-third of known inpatient cases in the state of Colorado as of April 30, 2020. However, other hospitals in the Denver metro area could have different statistics and populations.

In conclusion, we experienced a lower mortality (both total and ICU), ICU length of stay, ventilator days, ICU patients requiring intubation, and mechanical ventilation mortality of COVID-19 in the Denver area compared to other reports. The reasons for this are unclear but may be best explained by geographic and population-based factors.

## Figures and Tables

**Table 1:** Patient characteristics.

Patient characteristics	*n*	%
Included patients	681	—
Age, median (IQR) [range], y	64 (50–78) [0–103]	—
Sex		
Female	334	49.05
Male	347	50.95
Race		
White	341	50.07
Black	111	16.30
Others	169	24.82
Unknown	60	8.81
Comorbidities		
Patients with at least 1 comorbidity	534	78.41
Cancer	14	2.06
Cardiovascular disease		
Hypertension	265	38.91
Coronary artery disease	99	14.54
Congestive heart failure	107	15.71
Chronic respiratory disease		
Asthma	54	7.93
Chronic obstructive pulmonary disease	80	11.75
Obstructive sleep apnea	50	7.34
Immunosuppression		
History of solid organ transplant	4	0.59
Kidney disease	122	17.91
Liver disease	31	4.55
Metabolic disease		
Obesity (BMI ≥ 30)	154	22.61
Morbid obesity (BMI ≥ 35)	150	22.03
Diabetes	262	38.47
Smoking status		
Never smoker	375	55.07
Former smoker	104	15.27
Current smoker	30	4.41
Unknown	172	25.26
Total comorbidities		
None	147	21.59
1	164	24.08
>1	370	54.33
Total, median	2	

IQR = interquartile range; BMI = body mass index.

**Table 2:** Common comorbidities and outcomes.

	*n* (of total sample = 681)	Expired (*n*)	Mortality rate (%)	Required ICU (*n*)	ICU rate (%)	Required mech vent (*n*)	Vent rate (%)	Median LOS (days)
Male	347	48	13.83	118	34.01	68	19.60	5.08
Female	334	43	12.87	86	25.75	50	14.97	4.9
Never smoker	364	36	9.89	100	27.47	60	16.48	4.83
Former smoker	100	16	16.00	39	39.00	22	22.00	6.56
Current smoker	29	3	10.34	11	37.93	4	13.79	5.08
Nonobese	374	52	13.90	111	29.68	61	16.31	4.92
Obesity (BMI : 30–34.9)	154	20	12.99	45	29.22	28	18.18	4.9
Morbid obesity (BMI ≥ 35)	150	19	12.67	48	32.00	29	19.33	5.25
No comorbidities	147	3	2.04%^*∗*^	35	23.81	18	12.24	3.38
1 comorbidity	164	9	5.49%^*∗*^	44	26.83	26	15.85	4.19
>1 comorbidity	370	79	21.35%^*∗*^	125	33.78	74	20.00	6.08
Hypertension	265	35	13.21	87	32.83	51	19.25	5.21
Diabetes	262	62	23.66	102	38.93	64	24.43	6.71
COPD	80	18	22.50	32	40.00	19	23.75	8.27

*∗p* < 0.001.

**Table 3:** Discharge disposition and length of stay by age group.

Disposition by age group					
Age intervals, y	*n*	Expired, *n* (%)	LOS of expired, median days (IQR)	Discharged alive, *n* (%)	LOS of alive, median days (IQR)
0–18	2	0 (0)	N/A	2 (100)	4.65^*∗*^
19–30	30	1 (3.3)	8.17^*∗*^	29 (96.7)	3.38 (2.06–5.46)
31–64	318	17 (5.3)	8.21 (2.17–10.90)	301 (94.7)	4.08 (2.29–8.86)
65–84	233	48 (20.6)	8.13 (5.34–12.71)	185 (79.4)	5.21 (2.98–10.61)
85+	98	25 (25.5)	4.62 (2.61–6.42)	73 (74.5)	6.21 (3.23–14.36)

*∗*IQR omitted due to low *n*. LOS = length of stay.

**Table 4:** Outcomes and clinical measures.

Clinical measure	Total inpatients (*n* = 681)		Discharged alive				Expired			
	*n*	%	≤65 y (*n* = 332)	%	>65 y (*n* = 258)	%	18–65 y (*n* = 18)	%	>65 y (*n* = 73)	%
ICU care	204	29.96	87	26.20%	58	22.48%	16	88.89	43	58.90
Mechanical ventilation	118	17.33	48	14.46%	26	10.08%	14	77.78	35	47.95
Total deaths	91	13.36					18		73	
ICU deaths	59	8.66					16	88.89	43	58.90
Deaths on mechanical ventilation	49	7.20					14	77.78	35	47.95
Deaths without mechanical ventilation	42	6.17					4	22.22	38	52.05

Resource use
Length of stay, median days (IQR)	5.00 (2.79–9.83)		4.04 (2.29–8.24)		5.71 (3.00–10.96)		8.19 (2.37-10.32)		6.50 (3.96-10.61)	
			4.75 (2.75–9.76)				6.96 (3.96–10.29)			
ICU Length of stay, median days (IQR)	6.82 (2.56–12.27)		7.83 (3.00–13.54)		5.50 (1.79–10.41)		7.61 (1.52-10.72)		6.50 (3.00-9.62)	
			6.88 (2.40–13.36)				6.75 (7.00)			
Vent days, median days (IQR)	9.00 (5.00–13.00)		10 (7.00–12.75)		8.00 (5.00–15.00)		7.50 (4.00-10.50)		7.00 (4.00-10.00)	
			10.00 (6.00–14.00)				7.00 (6.00)			

Discharge disposition	*n*	%	*n*	%	*n*	%				
Total discharged alive	590	86.64	332	100.00	258	100.00				
Home	430	63.14	309	93.07	121	46.90				
Hospice	57	8.37	2	0.60	55	21.32				
Facilities	103	15.12	21	6.33	82	31.78				
Readmitted	86	12.63	18	5.42	68	26.36				

**Table 5:** Inpatient data comparison.

Outcome (with known discharge data)	Our hospitals in the Denver metro area	Buchwald et al. [11] (Colorado Prediction Model)	Zhou et al. [9] (Wuhan, China)	Richardson et al. [10] (NYC area, NY)	Grasselli et al. [8] (ICU, Lombardy, Italy)	Bhatraju et al. [7] (ICU, Seattle, WA)
Total number of patients	681	—	191	2634	—	—
Number of patients requiring ICU care	204 (30%)	(5–20%)	50 (26%)	373 (14.2%)	661	17
Number of ICU patients requiring invasive mechanical ventilation	118 (58%)	—	32 (64%)	320 (86%)	88%	13 (76%)

LOS (median days)						
Overall	5	8	11	4.1	—	—
ICU	6.82	10	8	Not reported	7–8	9
Mechanical ventilation days	9	—	Not reported	Not reported	Not reported	10

Mortality						
Overall	91/681 (13%)	—	54 (28%)	553 (21%)	—	—
ICU	59/204 (29%)	50%	Not reported	291 (78%)	405 (61%)	12 (71%)
Mechanical ventilation	49/118 (41.5%)	—	31 (97%)	282 (88.1%)	Not reported	9 (69%)

Readmissions	86 (12.6%)	—	Not reported	45 (1.7%)	Not reported	Not reported

## Data Availability

All data used to support the findings of this study are included within the tables.
